# An Anchor-Based Pedestrian Navigation Approach Using Only Inertial Sensors

**DOI:** 10.3390/s16030334

**Published:** 2016-03-07

**Authors:** Yang Gu, Qian Song, Yanghuan Li, Ming Ma, Zhimin Zhou

**Affiliations:** College of Electronics Science and Technology, National University of Defence Technology; Changsha 410073, Hunan, China; songqian@nudt.edu.cn (Q.S.); poptopli@163.com (Y.L.); maming19890911@163.com (M.M.); zm_nudt@163.com (Z.Z.)

**Keywords:** pedestrian navigation, anchor, Rao-Blackwellized particle filter, building structure

## Abstract

In inertial-based pedestrian navigation, anchors can effectively compensate the positioning errors originating from deviations of Inertial Measurement Units (IMUs), by putting constraints on pedestrians’ motions. However, these anchors often need to be deployed beforehand, which can greatly increase system complexity, rendering it unsuitable for emergency response missions. In this paper, we propose an anchor-based pedestrian navigation approach without any additional sensors. The anchors are defined as the intersection points of perpendicular corridors and are considered characteristics of building structures. In contrast to these real anchors, virtual anchors are extracted from the pedestrian’s trajectory and are considered as observations of real anchors, which can accordingly be regarded as inferred building structure characteristics. Then a Rao-Blackwellized particle filter (RBPF) is used to solve the joint estimation of positions (trajectory) and maps (anchors) problem. Compared with other building structure-based methods, our method has two advantages. The assumption on building structure is minimum and valid in most cases. Even if the assumption does not stand, the method will not lead to positioning failure. Several real-scenario experiments are conducted to validate the effectiveness and robustness of the proposed method.

## 1. Introduction

Self-contained inertial sensors have made foot-mounted pedestrian navigation an ideal way for positioning in emergency response missions under Global Positioning System (GPS)-denied environments, due to its independence from pre-installed infrastructures or predefined databases. With the adoption of Zero-velocity UPdaTe (ZUPT)-aided Extended Kalman Filter (EKF) algorithm, the cubic-in-time error growth for positioning is reduced to linear [1], thereby revealing the great potential of foot-mounted pedestrian navigation for practical use. However, many causes, including the deviations of low-cost Micro Electro Mechanical Systems (MEMS) Inertial Measurement Units (IMUs), especially the gyroscopes, can cause heading errors that accumulate over the long term, consequently resulting in positioning failure. There are three main reasons for heading error accumulation: (1) The Earth’s rotation. The turn rate of the local navigation frame with respect to the Earth’s frame is perceptible over the long term, reaching up to 15 degrees per hour. Furthermore, as many magnetic disturbances exist in an indoor environment [2], the direction of North can hardly be identified by a magnetometer. Therefore, the Earth’s rotation rate cannot justifiably be eliminated. (2) Measurement error. The angular rate measured by the gyroscopes suffers from different error sources, such as turn-to-turn random biases, instability over operation temperature range, *etc.* [3]. (3) Limitation of ZUPT-aided EKF algorithm. From an observability analysis of the ZUPT-aided EKF algorithm, roll and pitch errors are observable while the heading errors are not, demonstrating that the algorithm fails to bound heading errors [4]. Consequently, many methods are proposed to compensate the errors at a higher level than the trajectory-generation phase, namely the trajectory-calibration phase.

Anchors are effective for compensating the errors originating from the trajectory-generation phase, and are widely used in many trajectory-calibration methods. Traditional anchor-based methods can be divided into two types according to the *a priori* knowledge of the anchor map. If the anchor map is known beforehand, measurements from anchors can be directly adopted to calibrate trajectories; otherwise, the trajectory and the anchor map should both be estimated during the course of positioning, and it can be recognized as the Pedestrian Dead Reckoning (PDR) + Simultaneous Localization and Mapping (SLAM) problem. The first type includes methods relying on a predefined database such as Wireless Fidelity (WiFi) or magnetic fingerprints, to aid positioning [5,6]. Some other methods [7,8] use the densely deployed Ultra-Wide Bandwidth (UWB) and Radio Frequency IdentiFication (RFID) anchors to provide range from the anchors to the pedestrian, through Time of Arrival (ToA) and Received Signal Strength (RSS) measurements. The other type includes both the SemanticSLAM [9] and SmartSLAM method [10], which read unique points, like WiFi access points, in the surroundings as landmarks, combining them with PDR to form the SLAM framework. The SignalSLAM method [11] further adopts mixed signals as landmarks, including WiFi, Bluetooth, 4G Long Term Evolution (LTE) and magnetic signals.

Traditional anchor-based methods have remarkable performance in improving positioning accuracy. However, they either rely on an intact predefined database or on pre-installed infrastructures, such as WiFi access points and a UWB transceiver. Therefore, the system’s self-containedness is disrupted in these methods, and are thus not suitable for special applications, such as Search and Rescue (SAR), where self-containedness is crucial. From these anchor-based methods, we know that anchors are in essence additional information or measurements, to bound the raw trajectories from PDR. We expect to find a special anchor able to calibrate trajectories and still maintain the system’s self-containedness. Introducing *a priori* information of building structures as anchors could feasibly perform this task. The simplest way is to introduce floorplans of a building [12,13]; however, maps for buildings are not always available. Another method called Heuristic Drift Elimination (HDE) [14] adopts the partial information of the floorplan: the discrete domain directions. By assuming that the walking trajectories are generally aligned with these discrete directions, the heading drifts can be compensated. But in situations where the real heading significantly deviates from the domain directions (very common when the pedestrian walks complex paths, e.g., circles), the correction procedures may result in large positioning errors, due to the wrong heading estimation. Another method called Human behavior Aided Heading elimination (HAH) [15] has made some improvement on the HDE method, by combining human walking behavior with domain directions to increase accuracy. However, because it still relies heavily on pre-defined domain directions, it has the same limitation as the HDE method.

**Figure 1:**
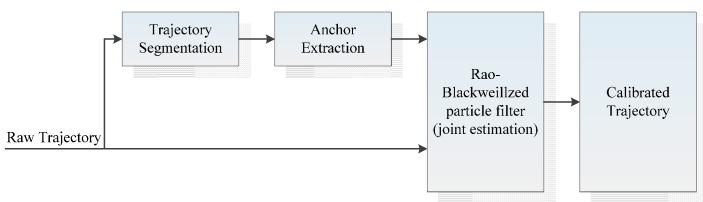
Overall framework of the proposed method.

The authors have proposed a “virtual” anchor-based approach for pedestrian navigation, which combines the notions of anchors with building structures. Compared to traditional anchor-based methods, it only relies on IMUs without any additional sensors or infrastructures. The “virtuality” denotes that anchors are extracted from the pedestrian’s trajectory in real time, and are considered to be the inferred characteristic of the building structures. Then a Rao-Blackwellized particle filter (RBPF) is adopted to perform anchor association (loop-closure detection) and position calibration. In our implementation, although the anchors are not distinguishable from each other according to their unique feature, we propose a new strategy to perform the anchor association, which awards the particles with associated anchors. This will be elaborated upon later in this paper. The experimental results have shown the effectiveness and robustness of the proposed method. Figure 1 presents the overall framework of the proposed method, where both the anchors and the trajectory are the inputs to the RBPF.

## 2. Anchor Definition and Extraction

### 2.1. Definition for Anchors

**Figure 2:**
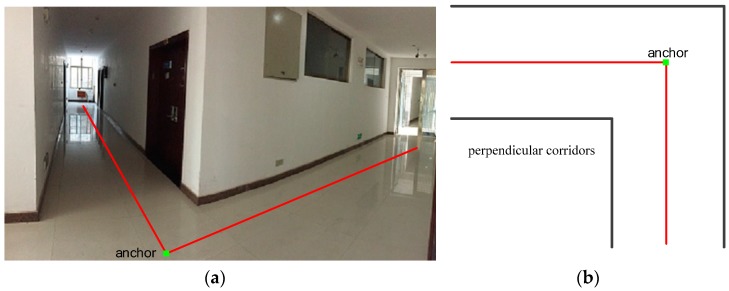
Anchor definition. (**a**) The real-scenario Perpendicular Intersectant Corridors (PIC) from a typical office building; (**b**) the floorplan of a PIC.

A conventional building with regular structure means that its corridors are either parallel or perpendicular to each other. Although some large buildings such as libraries or malls do not have such rectangular structures, they still have Perpendicular Intersectant Corridors (PICs). Figure 2a is an example of the real-scenario PICs, where the two corridors are perpendicular to each other and have an intersecting point. Figure 2b is the floorplan of the PIC. In our implementation, anchors are defined based on such structures. The two red lines lie in the middle of the corridors respectively, and their intersecting point is defined as an anchor.

Defining anchors in such a manner has three advantages. The anchors can reflect building structures and are time-invariant. It is therefore very helpful to compensate the time-varying trajectory errors. PIC structures are very common in buildings. Compared with domain directions used in the HDE method, the PICs are more applicable and not limited to rectangular buildings. The PIC structure can restrict a pedestrian’s trajectory to a great extent, including the headings and positions. As a result, a mapping relationship can be built between the PIC structure and a specific segment of trajectory. In other words, a real anchor (defined with the PIC structure) can be “observed” through trajectories, even without any extra sensors. The mapping of real anchors to virtual anchors (“observed” through trajectories) will be established in the next section. To avoid confusion, in our later description, we call the anchors defined with the PIC structure “real” anchors, and the anchors extracted from estimated trajectories “virtual” or “observed” anchors. Please note that wider corridors correspond to higher inaccuracy of anchor observation, thereby increasing the observation noise. This will be discussed in the measurement model.

### 2.2. Anchor Extraction from Trajectories

Without any additional sensors, anchors cannot be observed directly. However, virtual or observed anchors can be extracted from trajectories, and they can be considered as an observation of the real anchor with observation noise. Only with observations can an RBPF be used to solve the joint estimation of positions (trajectory) and maps (anchors) problem. The extraction or “observation” of anchors from the trajectory follows two steps: trajectory segmentation and anchor extraction from corridor-walks.

#### 2.2.1. Trajectory Segmentation

When a pedestrian walks along a corridor, a corridor-walk phase can be identified through the line segment of a trajectory. This is achieved through a trajectory segmentation scheme in [16] and is briefly reviewed here. The trajectory is partitioned into sub-trajectories according to their similarity to a straight-line segment. In our implementation, we want the corridor-walk segments to be extracted as accurately as possible. This can be achieved in two ways: The non-straight-line segments, such as turns, should be partitioned into several short straight-line segments, so that we can identify a corridor-walk according to the length of a partitioned sub-trajectory. Each partitioned segment should be as long as possible, so that minor deviations in a corridor-walk will not separate into several segments. They can also be expressed as the preciseness and conciseness property, respectively. In fact, the two properties of a certain partition pattern are contradictory. To find the optimal tradeoff between the two properties of a trajectory, a Minimum Description Length (MDL) principle is adopted, which is widely used in information theory.

Before forming the MDL principle, some definitions in the trajectory partitioning scheme are introduced. For the trajectory to be partitioned, it can be expressed as $TR=p_{1}p_{2}p_{3}...$, where the points $p_{1},p_{2},p_{3},...$ are sequentially connected to form the trajectory. If the trajectory is partitioned at point $p_{i}$, then it is defined as a characteristic point. The set of characteristic points can be denoted as $CP-\{p_{c1}p_{c2}p_{c3}...\}$ and they correspond to a specific partition pattern H.

**Figure 3:**
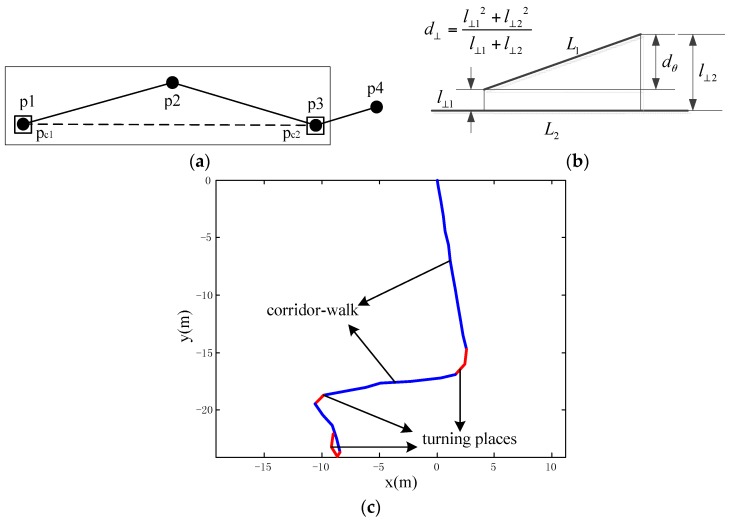
(**a**) A trajectory sample for MDL principle formation. $p_{c1}$ and $p_{c2}$ are characteristic points; (**b**) definition of the perpendicular distance $d_{⊥}$ and angle distances $d_{\theta }$ between two lines; (**c**) real-scenario trajectory partitioning result.

The MDL principle is then illustrated as follows: if the sum of preciseness and conciseness is minimum under partition pattern H, then H denotes the best partition of a trajectory. Preciseness is denoted by L(D|H), which measures the difference of the partitioned trajectory from a standard straight line. Conciseness is denoted by L(H), which is determined by the number of partitioned segments. For example, for Figure 3a, we assume that $p_{1}$ and $p_{3}$ are the characteristic points $p_{c1}$ and $p_{c2}$, and then L(H) and L(D|H) can be expressed in Equation (1). L(H) is the sum of the length for the partitioned segments, while L(D|H) denotes the sum of perpendicular and angle distances between two line segments. The expression $len(p_{1}p_{3})$ denotes the length of the straight line connecting the point $p_{1}$ with $p_{3}$. The perpendicular and angle distances are defined in Figure 2b, where $d_{⊥}()$ is the perpendicular distance and $d_{\theta }()$ is the angular distance.

(1) $$\begin{array}{l} L(H)=\log_{2}(len(p_{1}p_{3}))\\ L(D|H)=\log_{2}(d_{⊥}(p_{1}p_{2},p_{1}p_{3})+d_{⊥}(p_{2}p_{3},p_{1}p_{3}))\\ \;\;\;\;+\log_{2}(d_{\theta }(p_{1}p_{2},p_{1}p_{3})+d_{\theta }(p_{2}p_{3},p_{1}p_{3})) \end{array}$$

Under the previous definitions, the trajectory partitioning problem can be converted to minimizing the cost $C_{t}$ in Equation (2) according to the MDL principle. The partitioning process based on minimizing the cost can be performed in real time, if local optima are regarded as the global ones. This is described in detail in [17,18], and is therefore beyond the scope of this paper.

(2) $$C_{t}=L(H)+L(D|H)$$

Figure 3c is a real-scenario partitioning result of a pedestrian’s trajectory. To better demonstrate the result, adjacent partitioned segments are shown in different colors (red and blue). Because this scheme partitions trajectory according to its similarity to a straight line, when there are obvious turning places, the previous segment ends and a new segment starts, thereby decreasing L(D|H). On the other hand, L(H) increases with the number of trajectory partitions. Therefore, the minimizing scheme of the sum of L(D|H) and L(H) in Equation (2) can be intuitively regarded as partitioning trajectory into segments of straight lines, while keeping the numbers of partitions low. It thus follows that if a long straight-line segment is extracted, it can be regarded as a corridor-walk.

#### 2.2.2. Anchor Extraction from Corridor-Walks

**Figure 4:**
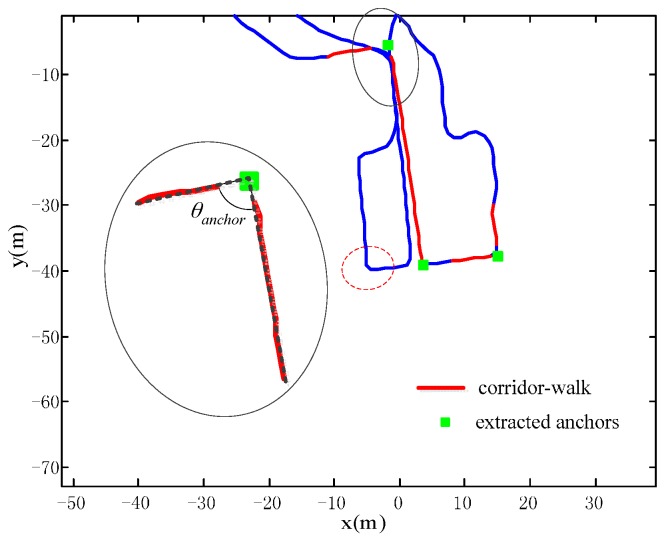
An example of anchor extraction from corridor-walks.

As illustrated previously, the anchors to be extracted from trajectories are not the intersecting points of the PIC structure in Figure 2b. They are “observations” of these points and are called virtual anchors, which have a one-to-one mapping relationship with real ones. They should have strong similarity to real anchors. An example of anchor extraction is shown in Figure 4. In the figure, three anchors are extracted from four corridor-walks (red segment), where the corridor-walks are identified through the trajectory segmentation scheme in Section 2.2.1. Please note that no anchors are extracted in the red dashed circle, because the partitioned line segments are not long enough to be deemed corridor-walks. Now we magnify the upper anchor for detail description. The anchor is extracted as the intersecting point of the extension lines (black dotted) of adjacent corridor-walks (red line segments). In addition, the adjacent corridor-walks should be very close to 90 degrees, to ensure the matching of virtual anchors and real ones. This condition is expressed in Equation (3), where $\theta_{anchor}$ denotes the angle between adjacent corridor-walks, and $\theta_{error}$ denotes the error tolerance from a right angle.

(3) $$\left|\theta_{anchor}-90\right|<\theta_{error}$$

**Figure 5:**
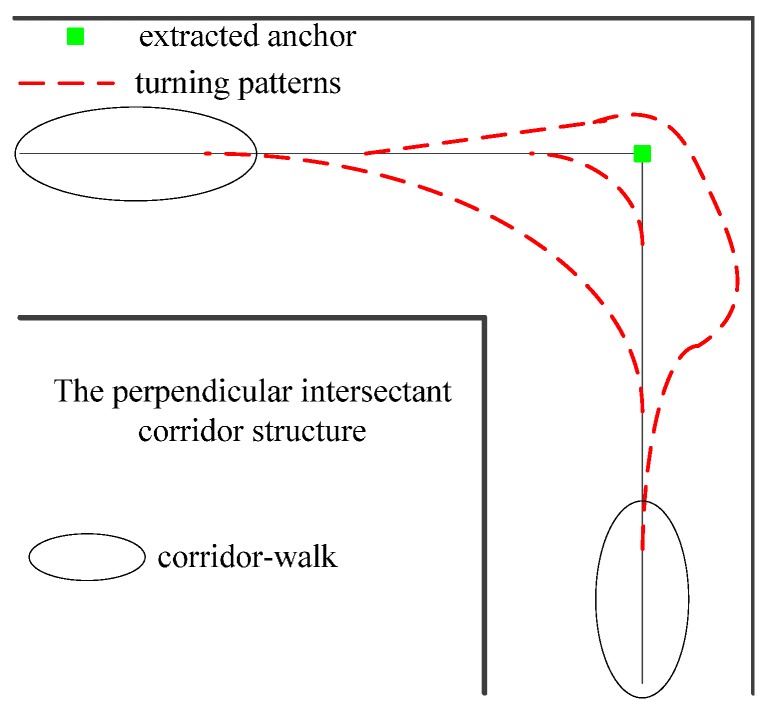
Anchor extraction in different turning patterns.

Extracting anchors in this manner has two advantages. The extracted anchors have strong similarities to the real ones. Because the extracted anchors are the intersecting points of the extension lines of adjacent corridor walks, they can represent the building structure, *i.e*., the anchors do not vary with the pedestrian’s walking behaviors. As shown in Figure 5, no matter how the pedestrian takes a turn (dashed red), the extension lines of the corridor-walks (in the ellipses) intersect at the extracted anchor (green square).

#### 2.2.3. Anchor Extraction Performance

**Figure 6:**
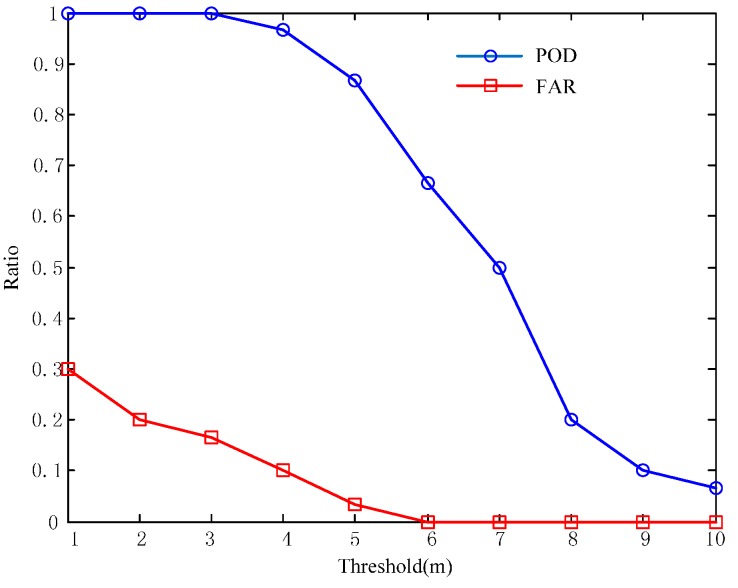
The Probability of Detection (POD) and the False Alarm Rate (FAR) curves. The *x*-axis denotes the threshold over which a line segment is identified as a corridor-walk.

As the proposed method adopts anchors to reduce positioning errors, it is important that the anchors are extracted correctly. Since correct anchor extraction is based on reliable identification of corridor-walks, close attention should be paid to it. We already have the general criterion for the identification of corridor-walks: If the length of the partitioned line segment is over a threshold, it can be regarded as a corridor-walk. However, how to choose the threshold remains a problem. Here we estimate the threshold based on two principles: the Probability of Detection (POD) should be as large as possible to make sure there are enough anchors in the SALM framework; the False Alarm Rate (FAR) should be as low as possible to increase the robustness of anchor detection. Figure 6 are the POD and FAR curves against the threshold of length of corridor-walks. The data set used here is a trajectory generated from a 30-min walk in a typical office building, where the true number of anchors can be acquired manually. From Figure 6, we can see that the two principles are in fact contradictory, because POD and FAR have positive correlations. We set the threshold at 5 m to reach a compromise, where POD is fairly high while the FAR is fairly low to make sure anchor detection is robust.

Because the anchor-extraction process is based only on the trajectory, if the trajectory fails to reveal the PIC structure, errors will appear in anchor extraction. More specifically, there are two such situations: (1) When the pedestrian walks so randomly (e.g., walks forward in circles in a PIC) that no corridor-walks are identified and the POD is very low; and (2) The pedestrian deliberately walks in a PIC-shaped trajectory without the existence of any PICs in open spaces, where false negative anchors can be extracted and the FAR is increased. However, these situations seldom occur in a pedestrian’s normal walks.

## 3. Rao-Blackwellized Particle Filtering Framework

By approximating real posterior density with finite samples, a particle filter can be used to perform nonlinear filtering, which is widely adopted in the field of navigation and positioning. A RBPF is a special particle filter that can make use of any linear Gaussian sub-structure to lower the dimensions of the state space to be estimated. Therefore, the number of particles needed to represent the state space is decreased [19]. In a SLAM problem, an RBPF can be used because the state space is naturally partitioned into the map and the position. In the field of robotics, the adoption of an RBPF can significantly decrease the number of particles needed and can convert the updating of maps to a simple linear Kalman filtering problem. Thus dramatically increasing the computation efficiency. Therefore, the adoption of the RBPF in a robotic SLAM problem is also called Fastslam [20].

Inspired by the Fastslam approach, we have proposed a new method to increase the accuracy of pedestrian navigation in the RBPF framework. In the framework, we define and extract anchors according to the easily identified PIC structures of buildings, which are considered as a coarse “map” in the SLAM problem. With the built pedestrian’s motion model and anchor observation model, the positioning problem is converted to a SLAM problem using an RBPF. Each particle has its own set of anchors (or “map”) and the anchor association is performed on a per-particle basis. For reweighting the particles, we have made a strategy that rewards the particles with more anchor association. In our implementation, these particles have better trajectory consistency (*i.e*., less error growth with time) at revisits. The experimental results have demonstrated the good performance in increasing the accuracy of pedestrian navigation.

### 3.1. Basics of the Filtering

From the perspective of the probabilistic description, the filtering is to estimate the posterior density $p(X_{t},M_{a}|z_{t},u_{t})$, where $X_{t}$ is the horizontal position vector of the pedestrian, $M_{a}$ denotes the anchor map, $z_{t}$ is the observation, $u_{t}$ is the driven vector for the pedestrian, and the suffix t indicates the tth step. Note that the filtering is step-wise, because the positions of the pedestrian are updated each time the pedestrian takes a step in the human motion model. If we ignore the anchor-involved states like $M_{a}$ and $z_{t}$, the posterior becomes $p(X_{t}|u_{t})$ and it is the motion model. Therefore, if no anchors are defined or used, the filtering results degenerate to the raw trajectory. To increase the location accuracy, we have merged the anchor-involved states $M_{a}$ and $z_{t}$ into the probabilistic model. On the surface, it seems that the redundant elements in the probabilistic model have increased the complexity of the filtering by adding the dimensions of state space to be estimated. However, if loop-closure happens and the anchors are correctly associated, *i.e.*, the anchors in the anchor map are re-observed, the correlation between the anchors would become increased and the joint probability density on all anchors $p(M_{a})$ would become peaked [21]. Note that the position vector $X_{t}$ is also correlated to the anchor map, so the uncertainty for the position estimation is diminished and the accuracy increased.

**Figure 7:**
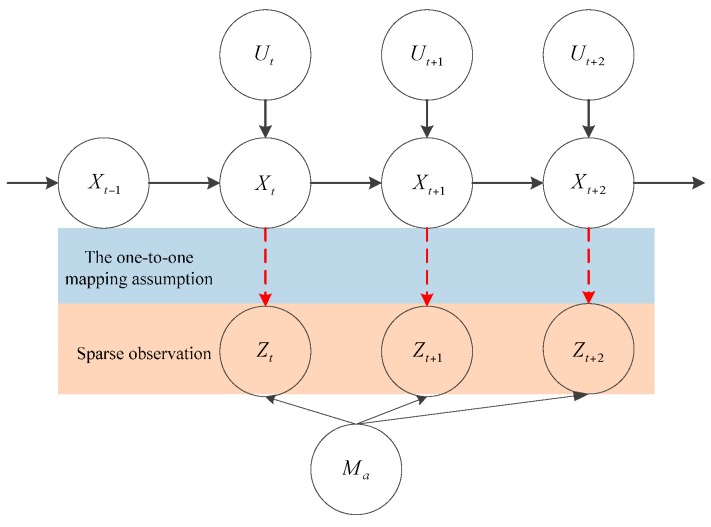
The generative probabilistic model for filtering.

As Figure 7 suggests, the generative probabilistic model of our approach is similar to that of a robotic SLAM problem. However, there are two unique features in the generative probabilistic model in our implementation. The observation may be very sparse or even non-existent. This may result from the reason that the pedestrian is walking in an unrestricted area and there are no PIC structures to form an anchor. However, this is very unlikely because the PIC structures are very common in buildings. Also, as the posterior density suggests, in the worst case, the positioning accuracy degenerates into that of the raw trajectory. The observation of anchors is under the assumption that it has a one-to-one mapping relationship with real anchors. This is not always true because the observation of anchors can also be extracted from a specific segment of trajectory in an open area. However, occasional wrong observation of anchors would not affect the positioning accuracy much, because they are highly unlikely to be re-observed. From the conditional dependence in Figure 7, the posterior $p(X_{t},M_{a}|z_{t},u_{t})$ can be factorized in Equation (4) similar to a SLAM problem, where the position update $p(X_{t}|z_{t},u_{t})$ is carried out in a particle filter and the map update $p(M_{a}|X_{t},z_{t},u_{t})$ is carried out in a Kalman filter.

(4) $$p(X_{t},M_{a}|z_{t},u_{t})=p(M_{a}|X_{t},z_{t},u_{t})p(X_{t}|z_{t},u_{t})$$

### 3.2. Motion Model

To recursively acquire particles sampled from $p(X_{t}|z_{t},u_{t})$, particles sampled from the proposal distribution $p(X_{t}|u_{t},{S^{\left[1;N\right]}}_{t-1})$ should be examined first. This proposal distribution is the motion model and ${S^{\left[1:N\right]}}_{t-1}$ is the particle set at time t−1. The driven vector $u_{t}$ is defined in a PDR (Pedestrian Dead Reckoning) way and is described in our previous work [15]. It is comprised of two components $\left\{L_{t},\Delta \theta_{t}\right\}$, where $L_{t}$ is the translation and $\Delta \theta_{t}$ is the heading change in one step. Thus the equation of the motion model can be written as:

(5) $$X_{t}=\left[\begin{array}{c} x_{t}\\ y_{t} \end{array}\right]=\left[\begin{array}{c} x_{t-1}\\ y_{t-1} \end{array}\right]+\left[\begin{array}{c} \left(L_{t}+L_{noise})\cos(\theta_{k-1}+\Delta \theta_{t}+\Delta \theta_{noise}\right)\\ \left(L_{t}+L_{noise})\sin(\theta_{k-1}+\Delta \theta_{t}+\Delta \theta_{noise}\right) \end{array}\right]=f(X_{t-1},u_{t},u_{noise})$$

In Equation (5), $\left\{x_{t},y_{t}\right\}$ is the position vector of the pedestrian and $u_{noise}$ can be considered as system noise. In our implementation, the proposal noise is chosen to be Gaussian and is proportional to the magnitude of $u_{t}$. This is based on the experience that greater translation $L_{t}$ and heading change $\Delta \theta_{t}$ generally means larger readings from the IMU, which will have larger measurement noise. Based on these facts, the noise particles are chosen in Equation (6). The coefficients a and b are chosen according to the performance of the inertial sensors, which is described in [15]. In this paper, we use the value 0.02 and 0.05 for a and b, respectively, and subtle change in the values is insignificant for the performance of the method.

(6) $$\begin{array}{c} {L^{\left[i\right]}}_{noise}\sim p(L_{noise})=\frac{1}{\sqrt{2\pi }\sigma_{L}}\exp(-\frac{{L_{noise}}^{2}}{2{\sigma_{L}}^{2}})\\ \Delta {\theta^{\left[i\right]}}_{noise}\sim p(\Delta \theta_{noise})=\frac{1}{\sqrt{2\pi }\sigma_{\Delta \theta }}\exp(-\frac{\Delta {\theta_{noise}}^{2}}{2{\sigma_{\Delta \theta }}^{2}})\\ \;{\sigma_{L}}^{2}=a\left|L_{t}\right|\\ \;{\sigma_{\Delta \theta }}^{2}=b\left|\Delta \theta_{t}\right| \end{array}$$

The new particle set ${S^{\left[1:N\right]}}_{t}$ is sampled from $p(X_{t}|u_{t},{S^{\left[1;N\right]}}_{t-1})$ and can be written as:

(7) $${S^{\left[i\right]}}_{t}=\left\{\begin{array}{c} {x^{\left[i\right]}}_{t}\\ {y^{\left[i\right]}}_{t}\\ {L^{\left[i\right]}}_{t}\\ \Delta {\theta^{\left[i\right]}}_{t} \end{array}\right\}=\left\{\begin{array}{c} {x^{\left[i\right]}}_{t-1}\\ {y^{\left[i\right]}}_{t-1}\\ {L^{\left[i\right]}}_{t-1}\\ \Delta {\theta^{\left[i\right]}}_{t-1} \end{array}\right\}+\left\{\begin{array}{c} {L^{\left[i\right]}}_{t}\cos(\theta_{k-1}+\Delta {\theta^{\left[i\right]}}_{t})\\ {L^{\left[i\right]}}_{t}\sin(\theta_{k-1}+\Delta {\theta^{\left[i\right]}}_{t})\\ {L^{\left[i\right]}}_{noise}\\ \Delta {\theta^{\left[i\right]}}_{noise} \end{array}\right\}$$

### 3.3. Measurement Model and Anchor Update

**Figure 8:**
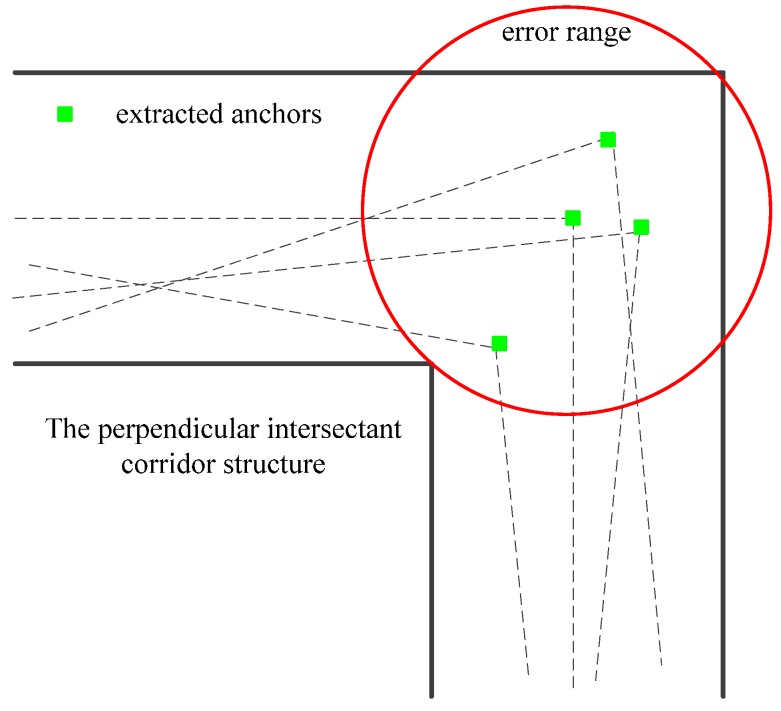
Noise assumption in the observation model.

As described in Section 2.2, the extraction of anchors is essentially an observation of anchors from the trajectories. Although observed anchors have a one-to-one mapping relationship with real anchors, observation noise still exists. Here we will describe the measurement model and analyze the noise. When an anchor is extracted from the raw trajectory, it means that there is an anchor in each trajectory hypothesis represented by the particle ${S^{\left[i\right]}}_{t}$. Thus the measurement model can be considered as the probabilistic distribution of the observed anchors conditioned on each particle $p(z_{t}|{S^{\left[i\right]}}_{t},L_{a})$, where $L_{a}$ is the estimated position of anchors. In our implementation, the observation is the horizontal position of the extracted anchors. As Figure 8 shows, because of the randomness of a pedestrian’s walking, the position of the extracted anchors is also random. For example, there are several possible anchors extracted from three different pairs of intersectant line segments in Figure 8. This randomness is regarded as observation noise and the noise is assumed to be 2-D Gaussian distributed for the purpose of similarity. As the anchor updating process is related to the covariance of the noise, the largeness of the noise should be ascertained. Here, the 3-σ ellipsoid is chosen to describe this largeness, where the percent probability of the extracted anchors lying in the 3-σ ellipsoid can reach up to 98.9%. In fact, the ellipsoid becomes a red circle and its radius is set to at 2 m, which matches the real scale of common corridors. The radius should be increased if the corridors are much wider. These assumptions and parameters are all made based on ground truth, and the influence of different settings will be studied in the future.

If a new extracted anchor is associated with a previously extracted one, the position of the anchor should be updated according to the observation. The updating can be written in a probabilistic form in Equation (8) and this can be carried out in a Kalman filter if we assume that the distributions are all Gaussian.

(8) $$\begin{array}{lll} p(L_{a}|X_{t},u_{t},z_{t}) & \overset{Bayes}{\propto } & p(z_{t}|L_{a},X_{t},z_{t-1},u_{t})p(L_{a}|X_{t},z_{t-1},u_{t})\\ & \overset{Markov}{=} & p(z_{t}|L_{a},X_{t})p(L_{a}|X_{t-1},z_{t-1},u_{t-1}) \end{array}$$

In this situation, the time update process of the Kalman filter is ignored, because we assume the estimated position of the anchor does not change until it is re-observed. We mainly focus on the measurement update part, which is proceeded each time the anchor is re-observed. Here we first discuss the measurement update when an anchor is observed the second time. It has three steps and the suffix is the number of times the anchor is observed.

1. Calculate the Kalman gain in Equation (10), where ${P_{2}}^{-}$, $H_{2}$ and $R_{2}$ are defined in Equation (9).
  (9) $${P_{2}}^{-}=\left[\begin{array}{cc} \sigma^{2} & 0\\ 0 & \sigma^{2} \end{array}\right],H_{2}=\left[\begin{array}{cc} 1 & 0\\ 0 & 1 \end{array}\right],R_{2}=\left[\begin{array}{cc} \sigma^{2} & 0\\ 0 & \sigma^{2} \end{array}\right]$$
  (10) $$\begin{array}{l} K_{2}={P_{2}}^{-}{H_{2}}^{T}{\left(H_{2}{P_{2}}^{-}{H_{2}}^{T}+R_{2}\right)}^{-1}\\ \;={P_{2}}^{-}{\left(2{P_{2}}^{-}\right)}^{-1}=1/2 \end{array}$$
2. Update the estimated position of an anchor ${\hat{L}}_{a}$ in Equation (12), where $K_{2}$ and $H_{2}$ are shown in Equation (11), noting that ${{\hat{L}}_{a}}^{-}$ is the previous estimated position of an anchor.
  (11) $$K_{2}=1/2,H_{2}=\left[\begin{array}{cc} 1 & 0\\ 0 & 1 \end{array}\right]$$
  (12) $$\begin{array}{l} {\hat{L}}_{a}={{\hat{L}}_{a}}^{-}+K_{2}(z_{2}-H_{2}{{\hat{L}}_{a}}^{-})\\ \;=1/2(z_{2}+{{\hat{L}}_{a}}^{-}) \end{array}$$
3. Update the covariance of the ${\hat{L}}_{a}$ in Equation (14), where $K_{2}$, $H_{2}$ and ${P_{2}}^{-}$ are shown in Equation (13).
  (13) $$K_{2}=1/2,H_{2}=\left[\begin{array}{cc} 1 & 0\\ 0 & 1 \end{array}\right],{P_{2}}^{-}=P_{1}$$
  (14) $$\begin{array}{l} P_{2}=(I-K_{2}H_{2}){P_{2}}^{-}\\ \;=1/2P_{1} \end{array}$$

This can be easily extended to the third time the same anchor is observed, or even afterwards. The results show that the more an anchor is observed, its error covariance decreases and further observations have a less significant influence on its estimation. This can be explained by the fact that the error of an inertial system grows with time when the observations originated from the inertial system.

### 3.4. Weight Update and Anchor Association

The anchor association problem is to associate the current extracted anchor with previously estimated anchors, which is carried out on a per-particle basis. Although it seems that an anchor extracted at a turning point does not have the unique feature needed to be distinguishable from other anchors, we can perform the anchor association based on the error models of the anchors and award the anchors matching the anchor map. The anchor association strategy can be described as follows.

The anchor association is based on the error range: if a previously estimated anchor’s position ${\hat{L}}_{a,t-1}$ is within the error range of the new observation $p(z_{t}|{S^{\left[i\right]}}_{t},L_{a})$, it is associated with the previous anchor and should be used to update the anchor position; otherwise the observation is assigned to a new anchor. Please note that the measurement model has already been outlined in Section 3.3, and the 3−σ ellipsoid is counted as the error range of an observation. The results of anchor association are related to the weight update process of the particle filter, which is specified as following.

**Figure 9:**
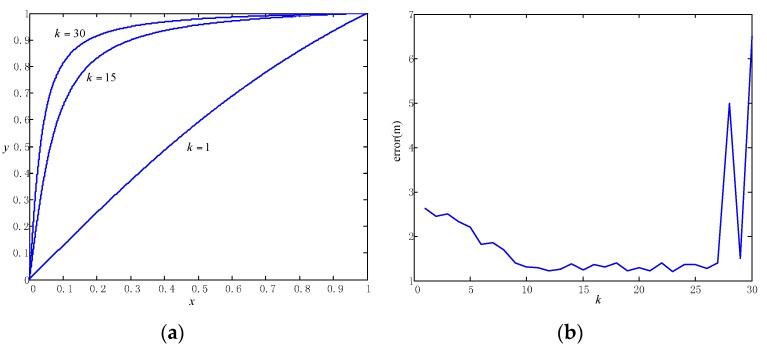
(**a**) The different values for the parameter k in the rewarding function correspond to different extents of convex; (**b**) the curve of positioning errors against different values of k.

In a RBPF, the importance weight of particles is gained in Equation (15). If we consider the anchor maps have a peaked posterior (previous anchors have less errors), then the importance weight is $p(z_{t}|{S_{t}}^{i},{\hat{L}}_{a,t})$. However, this is true only for the particles associated with the observation. For those unassociated anchors, they should also be assigned importance weight. We adopt a strategy to reward the associated particles in order to improve filter convergence and to ensure that anchor maps matching particles have more chances of “survival.” The rewarding function is written as y=r(x), where the input x is the percentage of the associated anchors and the output y is the sum of normalized importance weight for the associated anchors. The rewarding function should satisfy the two conditions: it should cross the point of (0,0) and (1,1); it should lie on the upper part of the line y=x and should be convex (to make sure the particles with associated anchors gain more weight). In fact, there are numerous such functions. Inspired by the excite functions commonly used in neural networks, we chose the function with the form y=marctan(kx), where m is a normalizer to make the function cross the point (1,1) and k denotes the extent of convex. We know that if the rewarding function is more convex, the less the particle weights are dispersed. In this way, the extent of convex will affect the rate of particle convergence. Based on that, we can guess that the final positioning accuracy is insensitive to the extent of convex. We also adopt real-scenario data to validate the estimation as shown in Figure 9.

From Figure 9a, we can see how the different values for the parameter k affect the extent of convex of the rewarding function. The extent of convex grows if k is larger. In Figure 9b, we can see that the final positioning error changes little when k varies from 10 to 25. It has validated our guess that the error is insensitive to the value of k within some range. We can also see that if k is too small, the reweighting of particles would be insignificant and the error would grow to the level of raw trajectory. If k is too large, the particles would be too concentrated, thereby destroying the diversity of particles and sometimes leading to large errors due to lack of robustness.

**Figure 10:**
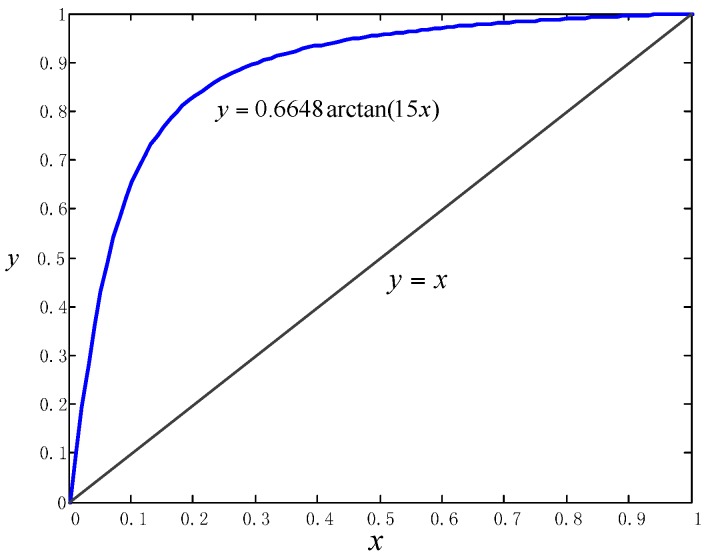
The chosen rewarding function for particle weight updating.

Overall, we chose the function y=0.6648arctan(15x) as shown in Figure 10, where k equals 15 according to the curve of Figure 9a and the normalizer m equals 0.6648 to make sure the rewarding function crosses the point (1,1). After the weighting process, the particles are resampled [22] to eliminate particles with small importance weights and duplicate particles with significant weights. Then the current position of the pedestrian is estimated with the average position of the largest group of particles with the same data association.

(15) $$\begin{array}{l} {w_{t}}^{i}=\int p(z_{t}|{S_{t}}^{i},M_{a})p(M_{a}|z_{1:t-1},{S_{1:t-1}}^{i})dM_{a}\\ \;\;\approx p(z_{t}|{S_{t}}^{i},{\hat{L}}_{a,t}),\;if\;p(M_{a}|z_{1:t-1},{S_{1:t-1}}^{i})\;is\;peaked \end{array}$$

## 4. Tests and Experiments

**Figure 11:**
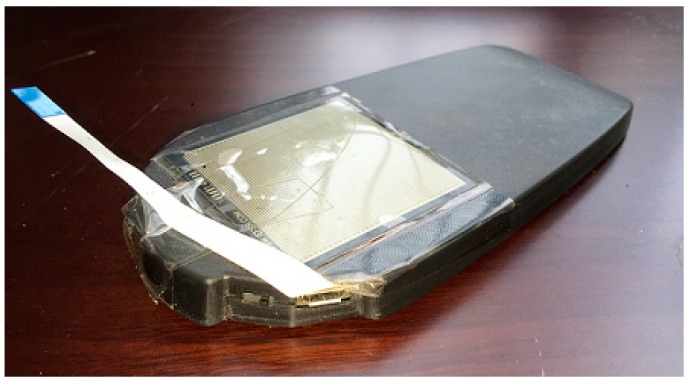
The insole-shaped MIMU (Multiple Inertial Measurement Unit) module.

All the raw trajectories are acquired from a module developed by the researchers. This module is a Multiple Inertial Measurement Unit (MIMU) platform with eight IMUs and has much lower measurement noise than a single IMU. The advantage of a MIMU platform has already been described in detail in [23], so it will not be discussed here. The module is also designed for convenient use. As shown in Figure 11, the PCB board is designed to fit in the insole-shaped shell, so that the module can be easily placed into shoes to conduct pedestrian positioning. The inertial measurements data are processed in real time using the classical ZUPT-aided EKF algorithm. The step-wise raw trajectory data is stored in a built-in flash memory and can be acquired from a USB interface.

**Figure 12:**
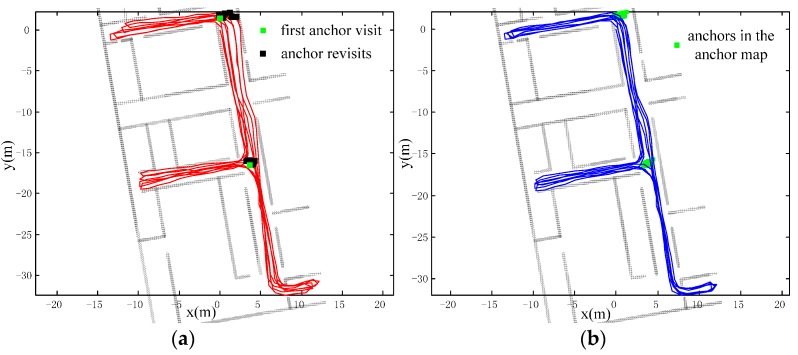
Test one: Office building scenario with abundant anchor revisits (**a**) Raw trajectory with multiple iterations; (**b**) calibrated trajectory with better consistency than raw trajectory.

**Figure 13:**
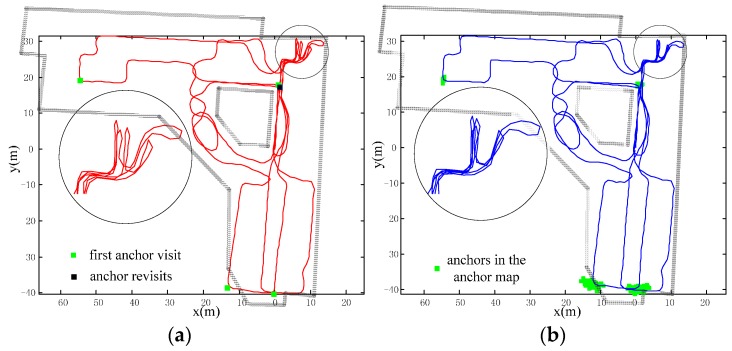
Test two: Library scenario with few anchor revisits (**a**) Raw trajectory with only one anchor revisit; (**b**) calibrated trajectory with better consistency in the circled area.

**Figure 14:**
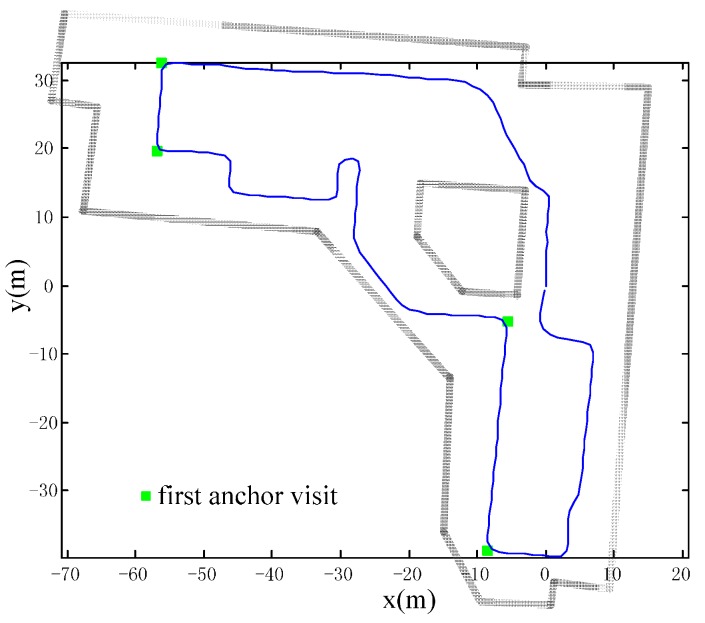
Test three: Library scenario with no anchor revisits. The accuracy of our method degenerates to the raw trajectory.

We have conducted three different real-scenario tests to demonstrate the effectiveness of our method in Figure 12, Figure 13 and Figure 14. They have diverse anchor revisit numbers and are depicted respectively. The trajectories in all tests have the same starting and ending point, so that the final positioning error can be easily calculated through the distance between the start and the position of the estimated ending point in the trajectories. As for the headings errors, they are calculated from the heading differences between the pre-defined true trajectory and the estimated trajectory. Such errors are often adopted to evaluate the positioning performance, as in [14]. We have also added the floor plan in the figures, to make the trajectory more meaningful.

1. The typical office scenario with many anchor revisits. This experiment is conducted in a typical office building and the trajectory has multiple iterations with a total length of about 523 m. There are abundant PIC walks and many revisits, so that a great deal of the anchor information can be adopted to calibrate the errors. As shown in Figure 12, the consistency of iterations of the calibrated trajectory (blue) are significantly higher than in the raw trajectory (red); *i.e*., heading errors are suppressed.
2. A library scenario with few anchor revisits is shown in Figure 13. There are many random walks with only partial iteration in the scenario. The total walking distance is about 729 m with only one anchor revisit (black dot) as shown in Figure 13a. It is hard to identify the true trajectory; however, we can tell the accuracy through the trajectory consistency of the area, which is purposely visited multiple times. From the upper right part of trajectory, we can see that our method has better consistency and has improved positioning accuracy.
3. A library scenario with no anchor revisits. Without any revisits of anchors, the positioning error cannot be calibrated. The accuracy will degenerate to raw trajectory as shown in Figure 14. The raw trajectory and the calibrated one coincide with each other (the blue trajectory). The results of this test may be obvious according to the principle of our approach; however, we still need the test to confirm the superiority over other domain direction assumption-based approaches, which will lead to positioning failure if the assumption does not stand.

Table 1 is a summary of quantitative results, including the heading error and the positioning errors (the percentage of positioning errors relative to the total travelled length is shown in the brackets) from the three tests, which shows that: with anchor revisits, the method is effective in suppressing both positioning and heading errors, even though there is only one revisited anchor; with no anchor revisits, the accuracy will degenerate to that of the raw trajectory (the minor difference is due to the randomness of noise added to the particles).

Our method is dependent on the structure of buildings. However, compared with other building structure-based methods, it has two remarkable advantages. The assumption on the building structure is minimum. Unlike the HDE methods, it does not need the condition that pre-defined domain directions exist in the whole building. It only needs the partial existence of the PICs, which is common in almost every building. The method will not lead to positioning failure when the assumption is not valid, and its accuracy at worst is the accuracy of raw trajectory. This advantage makes the method more flexible and robust than other building structure-based methods. We have conducted another experiment to demonstrate the improvements of our method over another building structure-based method [15] referred to as HAH, which combines eight pre-defined discrete domain direction and human walking behaviors to calibrate trajectories. Please note that the HDE method is not compared here, because it is not suitable for the random walk trajectory in our experiment.

**Figure 15:**
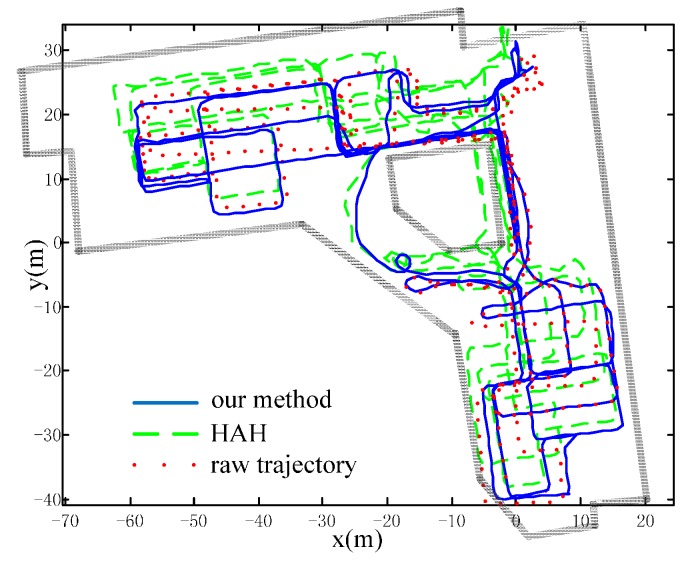
Trajectories for our method, the HAH method and raw trajectory. Our method has better accuracy than HAH and raw trajectory.

**Figure 16:**
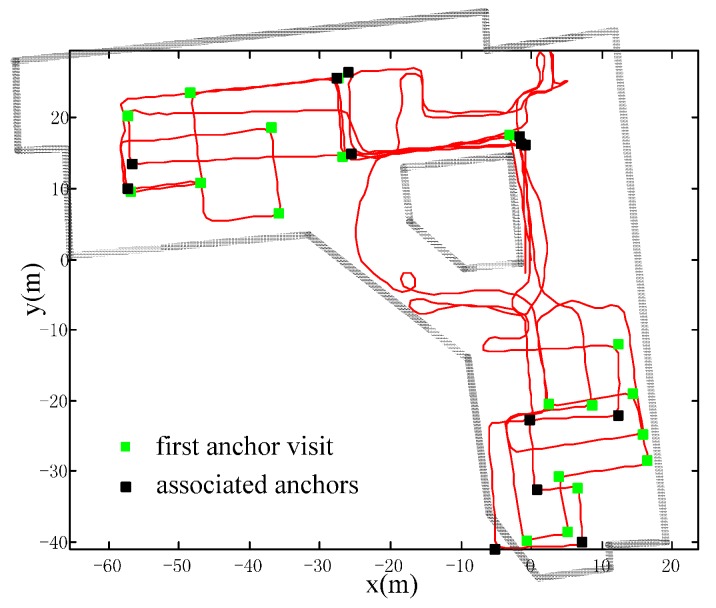
Raw trajectory and extracted anchors. The trajectory is designed to have intensive extracted anchors to test the robustness of the method when wrong anchor association is unavoidable.

The experiment has a total walking distance of about 995 m. The trajectories are compared in Figure 15 and the positioning errors are compared in Table 2. Our method has the best consistency and has lowered the error from about 2.0 m to 0.3 m, with the percentage of error relative to the distance traveled from 0.2% to 0.03%. The HAH method relies heavily on pre-defined directions, so that some sub-trajectories’ headings are falsely calibrated, especially in the areas where the trajectories are curves. Therefore, the error of HAH reaches up to about 7.5 m, even more than the error of the raw trajectory. This experiment can also demonstrate the robustness of our method. Figure 16 shows the raw trajectory and the extracted anchors (green ones denote first visited anchors; black ones denote their association with previous anchors). As we can see, the extracted anchors are intensive and the method sometimes has an incorrect anchor association. Because the pedestrian deliberately crossed the anchors with the ranges of a book shelf during the library walk, the extracted anchors are very close to each other and can lead to wrong anchor association. However, as the anchor association is based on a per-particle basis, partial wrong data association will not greatly affect the overall accuracy. In other words, this method is robust enough to cope with the intensive anchor situation, where wrong anchor association is unavoidable.

## 5. Conclusions

An anchor-based method for pedestrian navigation is proposed in this paper. In contrast to traditional methods, it does not rely on any extra infrastructures beside the IMUs. The anchors are extracted from the raw trajectory, according to the PIC structure of buildings. These anchors serve as observations and are fused into the Rao-Blackwellized particle filter framework. If revisits of anchors exist, both positioning and heading error are suppressed after the filtering.

The experiments have shown that our method is effective in calibrating trajectories, whether there are abundant revisits or very few. Moreover, this method is robust enough to cope with the obscure anchor problem, where extracted anchors are very close and inaccurate data association is unavoidable.

Compared with other building structure-based methods, advantages of our method are as follows: The assumption on building structure is minimum and valid in most cases. It only needs the existence of the PICs. Even if the assumption does not stand, the method will not lead to positioning failure, and the accuracy can still be kept the same as the raw trajectory at worst. However, the proposed method has the limitation that if no PICs exist in some highly irregular buildings, there would be no accuracy improvements over the raw trajectory. Solutions to this problem will be explored in future works through fusion information from other types of anchors.

## Figures and Tables

**Table 1:** Summary of the results from the tests with different amounts of anchor revisits.

Error Comparisons	Test 1		Test 2		Test 3	
	Heading Error	Positioning Error	Heading Error	Positioning Error	Heading Error	Positioning Error
Raw trajectory	0.14 rad	2.56 m (0.49%)	0.07 rad	1.35 m (0.19%)	0.05 rad	0.85 m (0.33%)
Our method	0.02 rad	1.34 m (0.25%)	0.03 rad	0.72 m (0.1%)	0.05 rad	0.87 m (0.34%)

**Table 2:** The positioning errors for raw trajectory, HAH method and our method.

Error Comparisons	Raw Trajectory	HAH	Our Method
Positioning error	2.0 m	7.5 m	0.3 m
Percentage of error relative to distance traveled	0.20%	0.75%	0.03%

## References

[1] Foxlin E. (2005). Pedestrian tracking with shoe-mounted inertial sensors. IEEE Comput. Graph. Appl..

[2] Li B., Gallagher T., Dempster A.G., Rizos C. How feasible is the use of magnetic field alone for indoor positioning?. Proceedings of the 2012 International Conference on Indoor Positioning and Indoor Navigation (IPIN).

[3] Woodman O.J. An Introduction to Inertial Navigation. https://acristoffers.me/pub-lic/CEFET/2%C2%BA%20Periodo/Introducao%20a%20Pratica%20Experimental/Referencias/10.1.1.63.7402.pdf.

[4] Nilsson J.O., Skog I., Händel P. Performance characterisation of foot-mounted ZUPT-aided INSs and other related systems. Proceedings of the 2010 International Conference on Indoor Positioning and Indoor Navigation (IPIN).

[5] Moreira A., Nicolau M.J., Meneses F., Costa A. Wi-Fi fingerprinting in the real world-RTLS@UM at the EvAAL competition. Proceedings of the 2015 International Conference on Indoor Positioning and Indoor Navigation (IPIN).

[6] Gozick B., Subbu K.P., Dantu R., Maeshiro T. (2011). Magnetic maps for indoor navigation. IEEE Trans. Instrum. Meas..

[7] Zampella F., Jimenez R., Antonio R., Seco F. Robust indoor positioning fusing PDR and RF technologies: The RFID and UWB case. Proceedings of the 2013 International Conference on Indoor Positioning and Indoor Navigation (IPIN).

[8] Ruiz A.R.J., Granja F.S., Honorato J., Rosas J.I.G. Pedestrian indoor navigation by aiding a foot-mounted IMU with RFID signal strength measurements. Proceedings of the 2010 International Conference on Indoor Positioning and Indoor Navigation (IPIN).

[9] Abdelnasser H., Mohamed R., Elgohary A., Farid M., Wang H., Sen S., Choudhury R.R., Youssef M. (2015). SemanticSLAM: Using Environment Landmarks for Unsupervised Indoor Localization. IEEE Trans. Mob. Comput..

[10] Shin H., Chon Y., Cha H. (2012). Unsupervised Construction of an Indoor Floor Plan Using a Smartphone. IEEE Trans. Syst. Man Cybern. Part C Appl. Rev..

[11] Nilsson M., Rantakokko J., Skoglund M.A., Hendeby G. Indoor positioning using multi-frequency RSS with foot-mounted INS. Proceedings of the 2014 International Conference on Indoor Positioning and Indoor Navigation (IPIN).

[12] Kaiser S., Khider M., Robertson P. (2011). A human motion model based on maps for navigation systems. EURASIP J. Wirel. Commun. Netw..

[13] Beauregard S., Klepal M. Indoor PDR performance enhancement using minimal map information and particle filters. Proceedings of the Position, Location and Navigation Symposium.

[14] Borenstein J., Ojeda L. (2010). Heuristic drift elimination for personnel tracking systems. J. Navig..

[15] Gu Y., Song Q., Li Y., Ma M. (2015). Foot-mounted Pedestrian Navigation based on Particle Filter with an Adaptive Weight Updating Strategy. J. Navig..

[16] Lee J.G., Han J., Li X. Trajectory outlier detection: A partition-and-detect framework. Proceedings of the 2008 ICDE IEEE 24th International Conference on Data Engineering.

[17] Lee J.G., Han J., Li X., Gonzalez H. (2008). TraClass: Trajectory classification using hierarchical region-based and trajectory-based clustering. Proc. VLDB Endow..

[18] Lee J.G., Han J., Whang K.Y. Trajectory clustering: A partition-and-group framework. Proceedings of the 2007 ACM SIGMOD International Conference on Management of Data.

[19] Grisetti G., Stachniss C., Burgard W. Improving grid-based slam with rao-blackwellized particle filters by adaptive proposals and selective resampling. Proceedings of the 2005 IEEE International Conference on Robotics and Automation.

[20] Montemerlo M., Thrun S., Koller D., Wegbreit B. FastSLAM: A Factored Solution to the Simultaneous Localization and Mapping Problem. http://www.aaai.org/Papers/AAAI/2002/AAAI02-089.pdf.

[21] Durrant-Whyte H., Bailey T. (2006). Simultaneous localization and mapping: Part I. IEEE Robot. Autom. Mag..

[22] Hol J.D., Schon T.B., Gustafsson F. (2006). On resampling algorithms for particle filters. Nonlinear Statistical Signal Processing Workshop.

[23] Skog I., Nilsson J.O., Handel P. Pedestrian tracking using an IMU array. Proceedings of the 2014 IEEE International Conference on Electronics, Computing and Communication Technologies (IEEE CONECCT).

